# The neuropathology of canine cerebral babesiosis compared to human cerebral malaria

**DOI:** 10.1186/1475-2875-13-S1-P55

**Published:** 2014-09-22

**Authors:** Andrew Leisewitz, Gareth Turner, Sarah Clift, Anne Pardini

**Affiliations:** 1 Department of Companion Animal Clinical Studies, Faculty of Veterinary Science, University of Pretoria, Pretoria, 0110, South Africa; 2 Mahidol Oxford Research Unit (MORU), Faculty of Tropical Medicine, Mahidol University, Bangkok 10400, Thailand; 3 Department of Paraclinical Sciences, Faculty of Veterinary Science, University of Pretoria, Pretoria, 0110, South Africa

## Background

The favoured animal model of cerebral malaria is an artificial host-parasite combination caused by *Plasmodium berghei* in inbred mouse strains [1]. Canine babesiosis caused by natural infection in dogs with *Babesia rossi* causes cerebral disease in some cases [2]. This disease demonstrates both similarities and differences to human malarial and comparisons may be of value in elucidating the pathogenesis of this serious complication in both hosts [3].

## Materials and methods

Post mortem brains collected from 50 natural cases of canine babesiosis showing clinical signs of cerebral involvement were collected and evaluated grossly and using light and electron microscopy.

## Results

Grossly visible lesions (seen in 31/50) were classified as global (16/50) or regional (34/50). Global lesions were diffuse swelling and diffuse cerebral congestion or pallor. Multifocal petechial haemorrhages and white matter malacia appeared more regional. There were 18/50 cases that had a grossly appreciable oedema. Histological lesions appeared in a spectrum of severity, and included very localized endothelial injury. Babesia-parasitised red cell sequestration was a feature in some sections. Early lesions were multifocal and strictly associated with the microvasculature. Intermediate lesions were characterized by perivascular haemorrhage and some neutrophil infiltration. Advanced lesions were locally extensive and similar in appearance to haemorrhagic infarction. Ultrastructural evidence of cytoadherence between erythrocytes and capillary endothelium was demonstrated. Endothelial cell necrosis occurred early in the development of the lesions before neuronal and glial changes.

## Conclusions

The endothelial injury, parasitized red cell packing and perivascular haemorrhage showed some similarities to the neuropathology of human CM. However the large haemorrhagic infarctions and clinical presentation with almost 100% mortality of dogs presenting with cerebral babesiosis were key differences.
